# Phase I Dose-Escalation Trial of an Innovative Chemotherapy Regimen Combining a Fractionated Dose of Irinotecan Plus Bevacizumab, Oxaliplatin, 5-Fluorouracil, and Folinic Acid (bFOLFIRINOX-3) in Chemorefractory Metastatic Colorectal Cancer

**DOI:** 10.3390/cancers13215472

**Published:** 2021-10-30

**Authors:** Hélène Bellio, Aurélie Bertaut, Alice Hervieu, Sylvie Zanetta, Audrey Hennequin, Julie Vincent, Rémi Palmier, Leila Bengrine-Lefevre, François Ghiringhelli, Jean-David Fumet

**Affiliations:** 1 Platform of Transfer in Biological Oncology, Georges François Leclerc Cancer Center—UNICANCER, 1 rue du Professeur Marion, 21000 Dijon, France; abertaut@cgfl.fr (A.B.); ahervieu@cgfl.fr (A.H.); szanetta@cgfl.fr (S.Z.); ahennequin@cgfl.fr (A.H.); jvincent@cgfl.fr (J.V.); rpalmier@cgfl.fr (R.P.); lbengrine@cgfl.fr (L.B.-L.); fghiringhelli@cgfl.fr (F.G.); 2 Department of Medical Oncology, Georges François Leclerc Cancer Center—UNICANCER, 1 rue du Professeur Marion, 21000 Dijon, France; 3 Maison de L’université Esplanade Erasme, University of Burgundy-Franche Comté, 21000 Dijon, France; 4 UMR INSERM 1231, 7 Boulevard Jeanne d’Arc, 21000 Dijon, France

**Keywords:** 5-fluorouracil, irinotecan, oxaliplatin, bevacizumab, chemotherapy metastatic colorectal cancer

## Abstract

**Simple Summary:**

Treatment of non-resectable metastatic colorectal cancer (mCRC) involves chemotherapy based on 5-fluorouracil, oxaliplatin and irinotecan and monoclonal antibodies targeting VEGF or EGFR. After an initial progression, it is usual to change the chemotherapy regimen and targeted therapy, with rather moderate results. Several studies have focused on the interest of using again already used molecules and rechallenge with oxaliplatin and irinotecan bi fractionation (FOLFIRI3) have previously shown efficacy in chemorefractory patients, but desynchronized triplet chemotherapy was never tested. The aim of this study was to evaluate the safety and efficacy of a new regimen so-called: FOLFIRINOX-3 bevacizumab in chemorefractory metastatic colorectal cancer.

**Abstract:**

The care of metastatic colorectal cancers is based on combination chemotherapies including 5-fluorouracil, oxaliplatin, irinotecan, and monoclonal antibodies targeting the epidermal growth factor receptor or vascular endothelial growth factor. The regimen is determined based on the patient’s molecular biology and general condition. Irinotecan bifractionation showed efficacy in chemorefractory patients in a previous study, FOLFIRI-3, but a desynchronized triplet has never been tested. The aim of bFOLFIRINOX-3 is to determine the safety, tolerance, and efficacy of a new regimen (FOLFIRINOX-3 bevacizumab) in chemorefractory patients. The aim of this study was to evaluate the safety and efficacy of FOLFIRINOX-3 bevacizumab in chemorefractory metastatic colorectal cancer (mCRC). A standard phase I, “3 + 3” design study was performed. The standard protocol comprised simplified FOLFOX 4 (folinic acid 400 mg/m^2^), 5-fluorouracil (a 400 mg/m^2^ bolus followed by 2400 mg/m^2^ for 46 h), oxaliplatin (85 mg/m^2^) and irinotecan (administered before and after 5-fluorouracil infusion), plus bevacizumab (5 mg/kg). In a “3 + 3” design, three different doses of irinotecan were tested: 60, 70 and 90 mg/m^2^. The primary endpoint was the maximum tolerable dose (MTD) of irinotecan. The secondary endpoints included the objective response (at 8 and 16 weeks) according to the RECIST 1.1 criteria and progression free survival. Thirteen patients were enrolled, and twelve patients were finally evaluated for dose-limiting toxicity (DLT). The dose level defined was 70 mg/m^2^ irinotecan. A total of three DLTs were observed (grade 3 diarrhea): two DLTs at the 90 mg/m^2^ dose level and one at the 70 mg/m^2^ dose level. The most frequently described adverse events were asthenia (93%), diarrhea (77%), nausea (62%) and peripheral sensory neuropathy (46%). The most frequent biological event was thrombopenia (54%). Regarding efficacy, among the 11 evaluable patients, no progression was observed at 8 weeks, and the partial response rate was 18.2%. At 16 weeks, a partial response rate of 27.3% was observed, and five patients had a stable disease. The new regimen of bFOLFIRINOX-3 with irinotecan at 70 mg/m^2^ was well tolerated. In chemorefractory patients, this protocol shows a high response rate.

## 1. Introduction

Colorectal cancer (CCR) is a common disease, with about 2 million new incident cases worldwide per year (GLOBOCAN 2021). It is the third most common cancer worldwide, and is responsible for approximately half a million deaths each year [1]. Approximately 30% of colorectal cancer patients have a synchronous metastatic disease [2,3], and about 40% of patients will develop metastases after surgery on the primary tumor [4].

When curative treatment is not feasible, metastatic or locally advanced colorectal cancers are treated with a first line combination of chemotherapies and targeted therapies, which have been shown to increase the rates of progression-free (PFS) and overall survival (OS) [5,6]. The most recent data for palliative chemotherapy show an overall survival of up to 30 months [7,8]. Palliative chemotherapy is currently based on various combinations of different chemotherapy molecules, combined with targeted anti-EGFR (Epidermal Growth Factor Receptor; panitumumab and cetuximab) or anti-VEGF (Vascular Endothelial Growth Factor; bevacizumab or aflibercept) monoclonal antibody therapies. The standard first-line treatments include fluorouracil (5-FU) and irinotecan (FOLFIRI), and/or oxaliplatin (mFOLFOX6) alone [9,10] or in combination with bevacizumab [5,6] or an anti-EGFR agent, according to the RAS and RAF status [11,12]. For patients with a good performance status, double or triple chemotherapies with target therapy in the first line have become the standard of care [13]. In recent years, triplet therapy, termed FOLFOXIRI plus bevacizumab, comprising the concurrent administration of fluorouracil, oxaliplatin, and irinotecan as a first-line therapy, has shown better antitumor activity than a doublet regimen using FOLFOX bevacizumab [14].

After the failure of 5-fluorouracil, oxaliplatin, irinotecan, and target therapies (bevacizumab, cetuximab, and panitumumab), few options are currently available [15,16]. Regorafenib, an oral multi-targeted tyrosine kinase inhibitor, has shown a modest improvement in OS compared with a placebo (alone), with a median overall survival of 6.4 months [17]. TAS-102 has also shown efficacy versus a placebo in this indication [18]. In a phase III study, TAS-102 yielded an overall survival of about 8 months [19]. However, the objective response rate remains very low, and the gain in terms of survival remains moderate. 

A rechallenge of previously used chemotherapies has also been described as a powerful strategy [20,21,22]. Oxaliplatin reintroduction or rechallenge yields PFS between 3 and 6 months, as reported in a recent systematic review [23]. Similarly, our group reported the efficacy of FOLFIRINOX chemotherapy (5-FU + folinic acid + irinotecan + oxaliplatin) in combination with bevacizumab as a rechallenge therapy in patients pretreated with FOLFOX and FOLFIRI [24]. Triple therapy could reverse chemotherapy resistance and yield a response rate of around 20%, a PFS of 6 months, and a survival of 12 months [24]. Another strategy is to optimize the FOLFIRI (5-FU + irinotecan) regimen. Irinotecan is an inhibitor of topoisomerase I, a helicase involved in DNA repair. Blocking topoisomerase when DNA damage is already present prevents the repair of these lesions and induces cell apoptosis. This rationale provides arguments in favor of the rapid re-administration of irinotecan. Indeed, preclinical data underline that re-administration with an interval of 3 days increases its efficacy [25]. Based on this rationale, the FOLFIRI regimen could be optimized by administering a half-dose of irinotecan on day 1 (before 5-FU) and a half-dose of irinotecan on day 3 (after 5-FU) in a desynchronized regimen called FOLFIRI3 [26]. The response rate was higher than that reported for a standard FOLFIRI-like regimen. Moreover, several previous studies have reported the benefit of re-challenge with oxaliplatin in the third or fourth line [27,28]. In the REOX study, after the reintroduction of oxaliplatin, almost 57% of the study patients showed disease control (complete + partial + stable disease) at twelve weeks, with a median time to treatment failure of 6 months in heavily pretreated patients [29]. The RE-OPEN study also assessed the effectiveness of reintroducing oxaliplatin in patients who had received prior chemotherapy including oxaliplatin and irinotecan, which achieved a response or stable disease, followed by confirmed disease progression ≥6 months previously during prior oxaliplatin-based therapy [28]. After twelve weeks of treatment, the disease control rate was 39.4% [28]. The addition of bevacizumab or aflibercept to FOLFIRI3 therapy also appears to improve its efficacy, with a PFS of 7 months [30].

However, the combination of oxaliplatin rechallenge with irinotecan desynchronization has never been tested. The objective of this study is thus to evaluate the safety of oxaliplatin rechallenge and irinotecan desynchronization using a new FOLFIRINOX-3 protocol in combination with bevacizumab in patients treated for metastatic colorectal cancer (mCRC) after 5 FU, oxaliplatin, and irinotecan.

## 2. Materials and Methods

### 2.1. Patient Selection

This phase I trial was an open-label study conducted at the Georges Francois Leclerc Centre, in Dijon, France. The trial was performed in accordance with the Declaration of Helsinki and Good Clinical Practice Guidelines. The study received approval from the Ethics Committee “CPP Sud-Est 1” (Saint Etienne, France) under the number 2018-38. The study was registered on ClinicalTrials.gov under the number NCT03795311, and with EudraCT under the number 2018-001452-36. All of the patients provided written informed consent before starting the trial.

Patients with advanced colorectal cancer who failed prior therapy by oxaliplatin, irinotecan, anti-VEGF and anti-EGFR (if indicated), or for whom no standard treatment options existed, were eligible for this study.

Patients aged 18 years or over were eligible if they had histologically confirmed mCRC; an Eastern Cooperative Oncology Group (ECOG) scale performance status (PS) of 0 or 1; were a fit for bFOLFIRINOX; and had an adequate hematological (absolute neutrophil count > 1:5.10^−9^/L, hemoglobin level > 9 g/dL, platelet count > 150.10^−9^/L), liver (total bilirubin level < 1.5 × upper limit normal (ULN), aspartate transaminase (AST) and alanine transaminase (ALT) < 5 × ULN, lactate dehydrogenase (LDH) < 5 × ULN) and renal (creatinine < 1.5 × ULN and calculated glomerular filtration rate (GFR) > 60 mL/min/1.73 m^2^) function. A tumor assessment was required at the baseline, with at least one measurable lesion according to the Response Evaluation Criteria in Solid Tumor (RECIST) version 1.1.

The exclusion criteria were the presence of any of the following: a diagnosis of additional malignancy within 5 years prior to inclusion (except curatively treated basal cell carcinoma of the skin and/or in situ cervical cancer); the presence of brain metastases; a life expectancy of less than 3 months; a medical contraindication incompatible with bevacizumab (major surgery during the last 28 days, a high risk of hemorrhage, a high risk of arterial thrombosis, or phlebitis without effective treatment); significant concomitant systemic disorders incompatible with the study; the demonstration of a dihydropyrimidine dehydrogenase (DPYD) and/or UDP-glucuronosyltransferase 1-1 (UGT1A) mutation (e.g., no evidence of homozygote UGT1A1*28 variants, and the plasma concentrations of uracil (U) should be lower than 16 ng/mL). Finally, pregnant and breastfeeding women were excluded from the study.

### 2.2. Treatment Plan and the Study Design

**Figure 1:**
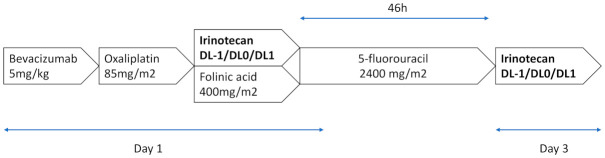
FOLFIRINOX 3–bevacizumab regimen. A treatment cycle consisted of 14 days of treatment. A maximum of 12 cycles was administered.

FOLFIRINOX was administered as per the standard procedures every 14 days, as follows: oxaliplatin 85 mg/m^2^ on day 1 as an intravenous (IV) infusion over 2 h, followed by folinic acid 400 mg/m^2^ as a 2 h IV infusion, with the addition of irinotecan 30, 35 or 45 mg/m^2^ according to the dose-level, given over 90-min IV, and followed by 5-fluorouracil 2400 mg/m^2^ as a continuous infusion over 46 h, without an IV bolus of 5-fluorouracil. This procedure was followed at the end of the 5-FU infusion by irinotecan 30, 35 or 45 mg/m^2^. Bevacizumab is administered every 14 days at a dose of 5 mg/kg as a 30-min IV infusion, before the oxaliplatin infusion (Figure 1).

The patients were planned to receive FOLFIRINOX 3–bevacizumab for a maximum of 12 cycles, until disease progression, unacceptable toxicity, or the patient refused to continue.

The patients were treated with increasing doses of irinotecan according to a “3 + 3 design”. Three dose levels (DLs) of irinotecan were planned (DL –1 to DL1): 60, 70 and 90 mg/m^2^. In brief, the inclusion started at DL0 (irinotecan 70 mg/m^2^). Two patients were enrolled. Dose limiting toxicities (DLTs) were observed for the first 2 cycles.

If no DLT was observed during the first 2 cycles, then one additional patient was treated at the same DL. If no DLT was observed for these first 3 patients, then the next 3 patients were included in a similar manner. If there was no DLT, or only one DLT was observed, then DL1 was investigated using the same model.

If one among the first three patients presented a DLT at any given DL, then three more patients had to be treated at the same DL. If a DLT occurred in this DL, then the dose escalation was fixed, and the next three patients enrolled into the treatment cohort were treated at the next lowest DL (e.g., DL–1). The unacceptable dose corresponded to the dose at which at least 33% (or 2/6) of the patients presented DLT.

DLT was defined as any of the following adverse events occurring during cycles 1 and 2: grade 4 thrombocytopenia or grade 3 thrombocytopenia associated with bleeding; febrile neutropenia; grade 4 diarrhea or grade 3 diarrhea without resolution after anti-diarrheal treatment; any drug-related non-hematological toxicity grade >3 (except alopecia, fatigue, nausea and adverse events during infusion quickly controlled by adaptive treatment).

A Data Safety Monitoring Board (DSMB) reviewed the results and safety data of this study, and prior approval from the DSMB was obtained before proceeding with the dose escalation.

### 2.3. Data Collection

For each cycle of FOLFIRINOX 3–bevacizumab administration, vital signs (body weight, temperature, blood pressure and heart rate (HR)), adverse events (AEs) and serious AEs (SAEs), a physical examination, and ECOG PS were collected on a regular basis, with a data cutoff 30 July 2021.

Every two weeks, a full biological assessment was performed, including a full blood count and complete biochemistry, renal function, and full liver function tests. Every two cycles, the laboratory evaluations also included angiotensin-converting enzyme (ACE) and carbohydrate antigen 19-9 (CA 19-9). 

For all of the patients, an electrocardiogram was performed at the baseline (prior to the first dose) and at the end of the study (a maximum of 14 days after the end).

The first tumor evaluation was performed at inclusion (prior to the first dose), and then every 8 weeks using the RECIST 1.1 criteria if there was at least one measurable lesion at the baseline.

Written informed consent was obtained from each patient before any screening or inclusion procedures. The patients remained on the study until one of the following conditions occurred: study withdrawal, treatment discontinuation, the study’s end, or death.

### 2.4. Statistical Analysis

A minimum of 12 and a maximum of 18 patients were to be included in phase I, and there was no statistical hypothesis to determine the sample size. All of the clinical and laboratory data are summarized by DL and overall. Safety analyses were performed on all of the patients enrolled and treated. The National Cancer Institute (NCI) Common Terminology Criteria for Adverse Events, version 4.03, was employed to graduate the AEs. All of the treatment-emergent AEs (TEAEs) and related TEAEs that were considered as probably, possibly or definitely related to FOLFIRINOX 3–bevacizumab are summarized in the tables, according to DL and overall.

## 3. Results

### 3.1. Patient Characteristics

A total of 13 patients were enrolled between 6 November 2018, and 16 December 2020. One of them was not included in the final analysis because the patient did not receive the second cycle of treatment, and DLT could not be evaluated. 

The study population comprised 13 patients, 54% females, with a median age of 63 years (range 40 to 74). The main tumor location was the right side (31%), followed by the sigmoid and rectum (23% each). Most (9/13, 70%) of the patients had KRAS mutations. All of the patients underwent surgery on the primary tumor, and six (46%) patients had received adjuvant chemotherapy in the past. The adjuvant chemotherapy regimen was FOLFOX in all six patients, and may explain why two patients did not receive oxaliplatin in the subsequent metastatic management. All of the patients previously received irinotecan and a target therapy for the treatment of metastatic disease.

The median time from the metastatic diagnosis to enrollment was 24 months, with a mean of 2.3 prior lines.

The patients’ characteristics are summarized in Table 1.

### 3.2. Dose-Limiting Toxicities

Twelve of the 13 treated patients were evaluable for DLT determination, and three DLTs occurred during the study (Table 2).

No DLT was observed for the first two patients at DL0, enabling the enrolment of one additional patient at DL0. One DLT (diarrhea) during cycle 1 was observed for patient 003 at DL0. Thus, three additional patients were treated at the same DL, with no further occurrence of any DLT.

According to the “3 + 3 design”, the next three patients were enrolled at DL1. One DLT (diarrhea) was observed for patient 009 during cycle 1. According to the protocol, three new patients were inserted at the same DL, but another DLT was observed in patient 013 (diarrhea) during cycle 2. At least 33% of the patients had a DLT at DL1, rendering this dose unacceptable.

One patient could not be evaluated for DLT due to a serious adverse event attributed to the treatment, grade 3 diarrhea, but this was not considered as a DLT because it lasted less than 3 days, with optimal symptomatic treatment. This 63-year-old female, diagnosed with left colorectal cancer and liver, lung, ovarian and peritoneal metastases, could not undergo cycle 2 due to the withdrawal of consent after this adverse event.

In conclusion, the dose level retained for phase II was DL0, i.e., 70 mg/m^2^.

### 3.3. Treatment-Related Adverse Events (TRAEs) and Biological Evaluation

TRAEs were reported in 13 (100%) patients, and all were related to the study drug. Among them, 11 were grade 3, and were related to FOLFIRINOX 3–bevacizumab, namely five diarrhea (two were not considered as DLT because they resolved quickly or happened after cycle 2), two high blood pressure, one peripheral sensory neuropathy, one anemia and two neutropenia. No grade 4 or 5 TRAEs were reported during the study treatment. One patient discontinued the study treatment due to a non-fatal adverse event (as described above) (Table 3).

The most frequently described TRAEs were as follows: asthenia (92%), diarrhea (77%), nausea (62%) and peripheral sensory neuropathy (46%), followed by thrombopenia (54%) and anemia (31%).

One patient presented a grade 2 hypersensitivity reaction during the oxaliplatin infusion at cycle 2. Allergic testing secondarily confirmed a hypersensitivity to oxaliplatin, constituting a contraindication to this treatment.

### 3.4. Antitumoral Activity

**Figure 2:**
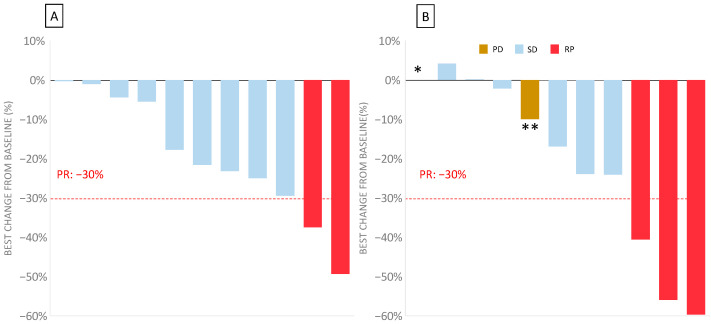
Waterfall plot of the disease evaluation in bFOLFIRINOX 3 measuring the maximum percentage change from the baseline for the target lesions for the 11 evaluable patients (two patients could not be evaluated for response) after 8 and 16 weeks. (**A**) Waterfall plot of the best change over the baseline at 8 weeks. (**B**) Waterfall plot of the best change over the baseline at 16 weeks. In one patient (*), the response was not evaluable because of their death before the next evaluation. In one patient (**), the evaluation according RECIST 1.1 showed an overall decrease but was classed as progressive disease due to the development of new lesions.

In this bFOLFIRINOX phase I trial, the best overall response in the 11 evaluable patients was a partial response in two patients (18.2%) at 8 weeks, with an additional nine patients with stable disease (81.8%) (Figure 2). No progressive disease was observed at 8 weeks. The response could not be evaluated in two patients: one due to toxicity (DLT) and one who had withdrawn from the study during cycle 1.

After 16 weeks, corresponding to the second assessment, three patients showed a partial response (27%), six patients had a stable disease (54%), and two patients had progressive disease (one death, and the other had a new suspect lesion).

**Figure 3:**
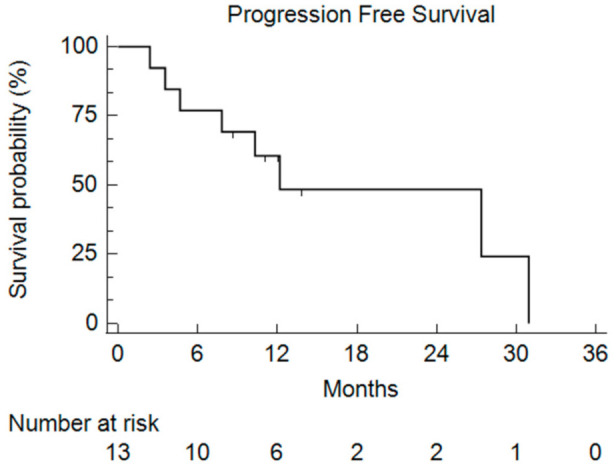
Kaplan–Meier curve of the Progression-Free Survival (PFS) for all of the enrolled patients who received bFOLFIRINOX.

After a median follow-up of 9.1 months, the median PFS was 11.1 months (2.4–30.9) (Figure 3).

## 4. Discussion

In this report, we presented the results of a phase I study that assessed the tolerability and efficacy of the FOLFIRINOX 3–bevacizumab regimen in patients with mCRC previously treated with standard therapies. Our results highlight the safety of this regimen in heavily pretreated patients, and support a recommended irinotecan dose of 70 mg/m^2^, given on days 1 and 3. The main toxic effect was diarrhea, present on average in 77% of the patients, of which 38.5% were grade 3. The dose escalation of irinotecan was limited by diarrhea toxicity in this phase 1 study during the DLT period, which was defined as the first 2 cycles of the FOLFIRINOX 3–bevacizumab regimen. These findings are unsurprising, and previous reports of FOLFIRI3 have described grade 3–4 diarrhea toxicity in around 30% of patients [30,31,32]. Other major adverse events included asthenia in 92% of cases, and grade 1–2 nausea/vomiting in 61.5%. Most of these side effects are classical with such chemotherapeutic regimens, and do not seem to be amplified in comparison with those observed using double or triple chemotherapies for mCRC. These adverse events were managed with routine supportive care according to the general practice guidelines [33].

Concerning hematotoxicity, 15.4% of our patients experienced grade 2 neutropenia, but no patient developed febrile neutropenia. The systematic use of pegfilgrastim support probably explains the few adverse events, contrasting with previous reports of grade 3–4 neutropenia with FOLFIRINOX in metastatic pancreatic cancer (45.7%), or in the TRIBE2 study with FOLFOXIRI-bevacizumab for mCRC (50%) [34]. There was no case of grade 3 thrombopenia in our study.

Until recently, after the first-line failure of fluorouracil, oxaliplatin, irinotecan and targeted therapies, very few treatments were available to improve patient outcomes. Most studies of patients in the third line of treatment report an overall survival of less than 6 months [35]. Recently, oral therapies, including TAS-102 and regorafenib, have shown a modest improvement in overall survival, with a survival gain of 2 months versus the placebo [17,19], although this treatment yields very few objective responses, and most patients who were initially stabilized by such a treatment rapidly failed. Thus, there is a place for more aggressive therapies for patients who still have a good performance status and a bulky disease that could rapidly become life-threatening. Our group previously reported a retrospective series of triplet chemotherapy (FOLFIRINOX bevacizumab) given in chemorefractory patients with a good performance status. The treatment compared favorably with oral monotherapy, with a median overall survival of 11.9 months and an overall response rate of 18% [24]. However, there may have been some potential for patient selection bias.

The efficacy of irinotecan in second- and third-line therapy is well described. The FOLFIRI3–bevacizumab protocol seems to provide a promising response rate, with 22% objective partial response in patients treated with one or two lines of chemotherapy [30]. The median survival seems similar to that observed with FOLFIRINOX, at around 12 months [30]. This study confirms the synergism between irinotecan and fluorouracil, with higher cytotoxicity when irinotecan is administrated after fluorouracil [36]. Thus, the desynchronization of irinotecan enables a decrease in toxicity. Moreover, re-challenge with oxaliplatin is now considered as a third or fourth line treatment option in patients with mCRC [27,28], especially in patients who experienced a response or stable disease for at least 6 months after prior oxaliplatin-based therapy.

In the present study, no progression was observed at 8 weeks, and there was a partial response rate of 18.2% (2/11). At 16 weeks, among the 11 evaluable patients, three showed a partial response (27% partial response rate) and five showed a stable disease. Only two disease progressions, including one death, were reported. PFS reached 11 months. The patients were selected based on their general condition (PS 0 or 1) and their good tolerance of previous chemotherapeutic regimens, with a high percentage of fit patients (77% PS 0). These selection criteria underline that the FOLFIRINOX3 protocol cannot be applied to a general population, which is often much more impaired after several lines of chemotherapy. The patients included in this phase I trial had received an average of 2.3 prior lines of chemotherapy, and half of them had received regorafenib or TAS-102 [37]. Although disease activity was not the main endpoint of the study, encouraging efficacy in heavily pretreated CRC patients was observed. These results must be tempered because of the low number of patients and the inherent patient selection bias observed in phase I clinical trials. In addition, the patients were strictly selected, with a lower median age than the general metastatic colorectal population and a highly preserved general condition [38]. In addition, with a median overall survival of 20 months in stage IV colorectal cancer patients [39], the patients in our study could be considered as being heavily pretreated, with a median time from metastatic diagnosis to enrollment of 24 months. However, these clinical results provide an efficacy signal that warrants confirmative studies.

## 5. Conclusions

This phase I dose-escalation study of bFOLFIRINOX-3 with irinotecan at a dose of 70 mg/m^2^ (i.e., 35 mg/m^2^ at day 1 and 35 mg/m^2^ at day 3) presented an acceptable toxicity profile and showed a very encouraging efficacy signal in the selected and fit patients. Indeed, the median PFS was 11.1 months after a median follow-up of 9.1 months in patients with a mean of 2.3 prior lines. A phase II study is ongoing to confirm these results.

## Figures and Tables

**Table 1:** Patient demographics and baseline characteristics.

Caracteristic	Total (N = 13)
Age (years)	
Median (SD)	63 (9.8)
Range	40–74
Sex (*n*; %)	
Female	7 (54)
Male	6 (46)
ECOG PS (*n*; %)	
0	10 (77)
1	3 (23)
Tumour location (*n*; %)	
Right side	4 (31)
Left side	2 (15)
Sigmoid	3 (23)
Rectum	3 (23)
Transverse	1 (8)
Initial stage	
Local	6 (46)
Metastatic	7 (54)
KRAS, NRAS, BRAF mutation (*n*; %)	
RAS and BRAF wild type	4 (30)
KRAS	9 (70)
NRAS	0 (0)
BRAF	0 (0)
Surgery on the primary tumor (*n*; %)	
Yes	13 (100)
Previous adjuvant chemotherapy *	
Yes	6 (46)
No	7 (54)
Previous therapy for metastatic colorectal cancer (*n*; %)	
Fluorouracil	13 (100)
Oxaliplatin	11 (85)
Irinotecan	13 (100)
Anti-EGFR therapy	4 (31)
Anti-VEGF therapy	10 (77)
Others **	5 (39)
Time from metastatic diagnosis to enrollment (days)	
Median (SD)	717 (544)
Range	(413–2338)

* Adjuvant chemotherapy was FOLFOX for all of the patients. ** Others included TAS-102 and regorafenib in monotherapy.

**Table 2:** Various irinotecan dose levels (DL) and dose-limiting toxicities (DLTs).

Irinotecan Dose Level, mg/m^2^	Total Number of Patients Treated	Patient Enrollment and Decision	Number of Patients with DLT
70	7	3 patients	1 DLT (diarrhea)
		3 additional patients	
90	6	6 patients	1 DLT (diarrhea)
			1 DLT (diarrhea)

DL, dose level; DLT, dose-limiting toxicity.

**Table 3:** Treatment-related adverse events (TRAEs) linked to FOLFIRINOX 3–BEVACIZUMAB (N = 13).

System Organ Disorder	All Grades	Grade 1	Grade 2	Grade 3
	N (%)	N (%)	N (%)	N (%)
Gastrointestinal disorders				
Diarrhea	10 (76.9)	3 (23.1)	2 (15.4)	5 (38.5)
Nausea	8 (61.5)	4 (30.8)	4 (30.8)	0 (0)
Anorexia	2 (15.4)	2 (15.4)	0 (0)	0 (0)
Mucitis	2 (15.4)	2 (15.4)	0 (0)	0 (0)
Nervous system disorders				
Peripheral sensory neuropathy	6 (46.1)	4 (30.8)	1 (7.7)	1 (7.7)
General disorders				
Asthenia	12 (92.3)	7 (53.8)	5 (38.5)	0 (0)
Vascular disorders				
Hypertension	2 (15.4)	0 (0)	0 (0)	2 (15.4)
Immune system disorders				
Allergic reaction	1 (7.7)	0 (0)	1 (7.7)	0 (0)
Skin and subcutaneous tissue disorders				
Palmar-plantar erythrodysestesia syndrome	1 (7.7)	0 (0)	1 (7.7)	0 (0)
Blood and lymphatic system disorders				
Anemia	4 (30.8)	2 (15.4)	1 (7.7)	1 (7.7)
Neutropenia	2 (15.4)	0 (0)	0 (0)	2 (15.4)
Thrombopenia	7 (53.8)	7 (53.8)	0 (0)	0 (0)
Liver function test disorders				
Alanine aminotransferase increased	1 (7.7)	1 (7.7)	0 (0)	0 (0)
Alkaline phosphatase increased	3 (23.1)	3 (23.1)	0 (0)	0 (0)
Metabolism and nutrition disorders				
Hypokalemia	1 (7.7)	0 (0)	1 (7.7)	0 (0)
Hypomagnesemia	1 (7.7)	1 (7.7)	0 (0)	0 (0)

E, event; N, number of patients.

## Data Availability

The data are available on request due to restrictions, e.g., privacy or ethics. The data presented in this study are available on request from the corresponding author. The data are not publicly available due to restrictions of privacy.

## References

[1] Parkin D.M., Bray F., Ferlay J., Pisani P. (2005). Global cancer statistics, 2002. CA Cancer J. Clin..

[2] Hackl C., Neumann P., Gerken M., Loss M., Klinkhammer-Schalke M., Schlitt H.J. (2014). Treatment of colorectal liver metastases in Germany: A ten-year population-based analysis of 5772 cases of primary colorectal adenocarcinoma. BMC Cancer.

[3] Engstrand J., Nilsson H., Stromberg C., Jonas E., Freedman J. (2018). Colorectal cancer liver metastases—A population-based study on incidence, management and survival. BMC Cancer.

[4] Jones R.P., Jackson R., Dunne D.F., Malik H.Z., Fenwick S.W., Poston G.J., Ghaneh P. (2012). Systematic review and meta-analysis of follow-up after hepatectomy for colorectal liver metastases. Br. J. Surg..

[5] Hurwitz H., Fehrenbacher L., Novotny W., Cartwright T., Hainsworth J., Heim W., Berlin J., Baron A., Griffing S., Holmgren E. (2004). Bevacizumab plus irinotecan, fluorouracil, and leucovorin for metastatic colorectal cancer. N. Engl. J. Med..

[6] Saltz L.B., Clarke S., Diaz-Rubio E., Scheithauer W., Figer A., Wong R., Koski S., Lichinitser M., Yang T.S., Rivera F. (2008). Bevacizumab in combination with oxaliplatin-based chemotherapy as first-line therapy in metastatic colorectal cancer: A randomized phase III study. J. Clin. Oncol..

[7] Douillard J.Y., Oliner K.S., Siena S., Tabernero J., Burkes R., Barugel M., Humblet Y., Bodoky G., Cunningham D., Jassem J. (2013). Panitumumab-FOLFOX4 treatment and RAS mutations in colorectal cancer. N. Engl. J. Med..

[8] Cremolini C., Loupakis F., Antoniotti C., Lupi C., Sensi E., Lonardi S., Mezi S., Tomasello G., Ronzoni M., Zaniboni A. (2015). FOLFOXIRI plus bevacizumab versus FOLFIRI plus bevacizumab as first-line treatment of patients with metastatic colorectal cancer: Updated overall survival and molecular subgroup analyses of the open-label, phase 3 TRIBE study. Lancet Oncol..

[9] Tournigand C., Andre T., Achille E., Lledo G., Flesh M., Mery-Mignard D., Quinaux E., Couteau C., Buyse M., Ganem G. (2004). FOLFIRI followed by FOLFOX6 or the reverse sequence in advanced colorectal cancer: A randomized GERCOR study. J. Clin. Oncol..

[10] Tournigand C., Cervantes A., Figer A., Lledo G., Flesch M., Buyse M., Mineur L., Carola E., Etienne P.L., Rivera F. (2006). OPTIMOX1: A randomized study of FOLFOX4 or FOLFOX7 with oxaliplatin in a stop-and-Go fashion in advanced colorectal cancer—A GERCOR study. J. Clin. Oncol..

[11] Bokemeyer C., Bondarenko I., Hartmann J.T., de Braud F., Schuch G., Zubel A., Celik I., Schlichting M., Koralewski P. (2011). Efficacy according to biomarker status of cetuximab plus FOLFOX-4 as first-line treatment for metastatic colorectal cancer: The OPUS study. Ann. Oncol..

[12] Van Cutsem E., Kohne C.H., Hitre E., Zaluski J., Chang Chien C.R., Makhson A., D’Haens G., Pinter T., Lim R., Bodoky G. (2009). Cetuximab and chemotherapy as initial treatment for metastatic colorectal cancer. N. Engl. J. Med..

[13] Van Cutsem E., Cervantes A., Nordlinger B., Arnold D., Group E.G.W. (2014). Metastatic colorectal cancer: ESMO Clinical Practice Guidelines for diagnosis, treatment and follow-up. Ann. Oncol..

[14] Falcone A., Ricci S., Brunetti I., Pfanner E., Allegrini G., Barbara C., Crino L., Benedetti G., Evangelista W., Fanchini L. (2007). Phase III trial of infusional fluorouracil, leucovorin, oxaliplatin, and irinotecan (FOLFOXIRI) compared with infusional fluorouracil, leucovorin, and irinotecan (FOLFIRI) as first-line treatment for metastatic colorectal cancer: The Gruppo Oncologico Nord Ovest. J. Clin. Oncol..

[15] Kim T.W., Elme A., Kusic Z., Park J.O., Udrea A.A., Kim S.Y., Ahn J.B., Valencia R.V., Krishnan S., Bilic A. (2016). A phase 3 trial evaluating panitumumab plus best supportive care vs best supportive care in chemorefractory wild-type KRAS or RAS metastatic colorectal cancer. Br. J. Cancer.

[16] Van Cutsem E., Peeters M., Siena S., Humblet Y., Hendlisz A., Neyns B., Canon J.L., Van Laethem J.L., Maurel J., Richardson G. (2007). Open-label phase III trial of panitumumab plus best supportive care compared with best supportive care alone in patients with chemotherapy-refractory metastatic colorectal cancer. J. Clin. Oncol..

[17] Grothey A., Van Cutsem E., Sobrero A., Siena S., Falcone A., Ychou M., Humblet Y., Bouche O., Mineur L., Barone C. (2013). Regorafenib monotherapy for previously treated metastatic colorectal cancer (CORRECT): An international, multicentre, randomised, placebo-controlled, phase 3 trial. Lancet.

[18] Mayer R.J., Van Cutsem E., Falcone A., Yoshino T., Garcia-Carbonero R., Mizunuma N., Yamazaki K., Shimada Y., Tabernero J., Komatsu Y. (2015). Randomized trial of TAS-102 for refractory metastatic colorectal cancer. N. Engl. J. Med..

[19] Xu J., Kim T.W., Shen L., Sriuranpong V., Pan H., Xu R., Guo W., Han S.W., Liu T., Park Y.S. (2018). Results of a Randomized, Double-Blind, Placebo-Controlled, Phase III Trial of Trifluridine/Tipiracil (TAS-102) Monotherapy in Asian Patients With Previously Treated Metastatic Colorectal Cancer: The TERRA Study. J. Clin. Oncol..

[20] Peixoto R.D., Kumar A., Lim H.J. (2015). Palliative oxaliplatin-based chemotherapy after exposure to oxaliplatin in the adjuvant setting for colon cancer. J. Gastrointest. Oncol..

[21] Tonini G., Imperatori M., Vincenzi B., Frezza A.M., Santini D. (2013). Rechallenge therapy and treatment holiday: Different strategies in management of metastatic colorectal cancer. J. Exp. Clin. Cancer Res..

[22] Townsend A.R., Bishnoi S., Broadbridge V., Beeke C., Karapetis C.S., Jain K., Luke C., Padbury R., Price T.J. (2013). Rechallenge with oxaliplatin and fluoropyrimidine for metastatic colorectal carcinoma after prior therapy. Am. J. Clin. Oncol..

[23] Mauri G., Gori V., Bonazzina E., Amatu A., Tosi F., Bencardino K., Ruggieri L., Patelli G., Arena S., Bardelli A. (2020). Oxaliplatin retreatment in metastatic colorectal cancer: Systematic review and future research opportunities. Cancer Treat. Rev..

[24] Chaix M., Vincent J., Lorgis V., Ghiringhelli F. (2014). FOLFIRINOX bevacizumab is a promising therapy for chemorefractory metastatic colorectal cancer. Oncology.

[25] Mullany S., Svingen P.A., Kaufmann S.H., Erlichman C. (1998). Effect of adding the topoisomerase I poison 7-ethyl-10-hydroxycamptothecin (SN-38) to 5-fluorouracil and folinic acid in HCT-8 cells: Elevated dTTP pools and enhanced cytotoxicity. Cancer Chemother. Pharmacol..

[26] Mabro M., Artru P., Andre T., Flesch M., Maindrault-Goebel F., Landi B., Lledo G., Plantade A., Louvet C., de Gramont A. (2006). A phase II study of FOLFIRI-3 (double infusion of irinotecan combined with LV5FU) after FOLFOX in advanced colorectal cancer patients. Br. J. Cancer.

[27] Chambers A.E., Frick J., Tanner N., Gerkin R., Kundranda M., Dragovich T. (2018). Chemotherapy re-challenge response rate in metastatic colorectal cancer. J. Gastrointest. Oncol..

[28] Suenaga M., Mizunuma N., Matsusaka S., Shinozaki E., Ozaka M., Ogura M., Yamaguchi T. (2015). Phase II study of reintroduction of oxaliplatin for advanced colorectal cancer in patients previously treated with oxaliplatin and irinotecan: RE-OPEN study. Drug Des. Devel. Ther..

[29] Costa T., Nunez J., Felismino T., Boente L., Mello C. (2017). REOX: Evaluation of the Efficacy of Retreatment With an Oxaliplatin-containing Regimen in Metastatic Colorectal Cancer: A Retrospective Single-center Study. Clin. Colorectal Cancer.

[30] Ghiringhelli F., Vincent J., Guiu B., Chauffert B., Ladoire S. (2012). Bevacizumab plus FOLFIRI-3 in chemotherapy-refractory patients with metastatic colorectal cancer in the era of biotherapies. Investig. New Drugs.

[31] Carola C., Ghiringhelli F., Kim S., Andre T., Barlet J., Bengrine-Lefevre L., Marijon H., Garcia-Larnicol M.L., Borg C., Dainese L. (2018). FOLFIRI3-aflibercept in previously treated patients with metastatic colorectal cancer. World J. Clin. Oncol..

[32] Kim S., Dobi E., Jary M., Monnien F., Curtit E., Nguyen T., Lakkis Z., Heyd B., Fratte S., Cleau D. (2013). Bifractionated CPT-11 with LV5FU2 infusion (FOLFIRI-3) in combination with bevacizumab: Clinical outcomes in first-line metastatic colorectal cancers according to plasma angiopoietin-2 levels. BMC Cancer.

[33] Fox P., Darley A., Furlong E., Miaskowski C., Patiraki E., Armes J., Ream E., Papadopoulou C., McCann L., Kearney N. (2017). The assessment and management of chemotherapy-related toxicities in patients with breast cancer, colorectal cancer, and Hodgkin’s and non-Hodgkin’s lymphomas: A scoping review. Eur. J. Oncol. Nurs..

[34] Eraslan E., Yildiz F., Tufan G., Aslan F., Demirci U., Oksuzoglu O.B. (2019). First line modified Folfirinox versus gemcitabine for advanced pancreatic cancer: A single institution retrospective experience. J. Oncol. Sci..

[35] Walter T., Hawkins N.S., Pollock R.F., Colaone F., Shergill S., Ross P.J. (2020). Systematic review and network meta-analyses of third-line treatments for metastatic colorectal cancer. J. Cancer Res. Clin. Oncol..

[36] Falcone A., Di Paolo A., Masi G., Allegrini G., Danesi R., Lencioni M., Pfanner E., Comis S., Del Tacca M., Conte P. (2001). Sequence effect of irinotecan and fluorouracil treatment on pharmacokinetics and toxicity in chemotherapy-naive metastatic colorectal cancer patients. J. Clin. Oncol..

[37] Tampellini M., Di Maio M., Baratelli C., Anania L., Brizzi M.P., Sonetto C., La Salvia A., Scagliotti G.V. (2017). Treatment of Patients With Metastatic Colorectal Cancer in a Real-World Scenario: Probability of Receiving Second and Further Lines of Therapy and Description of Clinical Benefit. Clin. Colorectal Cancer.

[38] Siegel R.L., Miller K.D., Goding Sauer A., Fedewa S.A., Butterly L.F., Anderson J.C., Cercek A., Smith R.A., Jemal A. (2020). Colorectal cancer statistics, 2020. CA Cancer J. Clin..

[39] Liu Z., Xu Y., Xu G., Baklaushev V.P., Chekhonin V.P., Peltzer K., Ma W., Wang X., Wang G., Zhang C. (2021). Nomogram for predicting overall survival in colorectal cancer with distant metastasis. BMC Gastroenterol..

